# Treatment outcomes of daptomycin- and vancomycin-resistant *Enterococcus faecium* bloodstream infection

**DOI:** 10.1093/jacamr/dlag108

**Published:** 2026-06-12

**Authors:** Wei-Ting Lin, Jia-Ling Yang, Chi-Ying Lin, Sung-Hsi Huang, Yu-Chung Chuang, Jann-Tay Wang, Yee-Chun Chen, Shan-Chwen Chang

**Affiliations:** Department of Internal Medicine, National Taiwan University Hospital, 7, Zhongshan S. Rd., Zhongzheng Dist., Taipei City 100225, Taiwan; Department of Internal Medicine, National Taiwan University Hospital, 7, Zhongshan S. Rd., Zhongzheng Dist., Taipei City 100225, Taiwan; Department of Internal Medicine, National Taiwan University Hospital Yun-Lin Branch, Yun-Lin County 640203, Taiwan; Department of Internal Medicine, National Taiwan University Hospital Hsin-Chu Branch, Hsin-Chu City 300195, Taiwan; Department of Internal Medicine, National Taiwan University Hospital, 7, Zhongshan S. Rd., Zhongzheng Dist., Taipei City 100225, Taiwan; Department of Internal Medicine, National Taiwan University Hospital, 7, Zhongshan S. Rd., Zhongzheng Dist., Taipei City 100225, Taiwan; Department of Internal Medicine, National Taiwan University Hospital, 7, Zhongshan S. Rd., Zhongzheng Dist., Taipei City 100225, Taiwan; Department of Internal Medicine, National Taiwan University Hospital, 7, Zhongshan S. Rd., Zhongzheng Dist., Taipei City 100225, Taiwan

## Abstract

**Objectives:**

Among patients with daptomycin-resistant and vancomycin-resistant *Enterococcus faecium* bloodstream infection (BSI), the optimal definitive therapy is uncertain. We estimated the dose effect of daptomycin (≥11 mg/kg versus 8 to <11 mg/kg) on 28-day mortality and exploratorily described outcomes in patients treated with linezolid.

**Methods:**

Adults with VRE BSI who received linezolid or daptomycin ≥8 mg/kg were included. *Post hoc* daptomycin MICs were determined by Sensititre broth microdilution (BMD), and episodes with MIC ≥8 mg/L were analyzed. The primary outcome was 28-day in-hospital mortality.

**Results:**

Among 130 patients with daptomycin-resistant VRE BSI, 110 received daptomycin and 20 received linezolid. Within the daptomycin group, 28-day mortality was 55.1% (43/78) with daptomycin 8 to <11 mg/kg and 34.4% (11/32) with daptomycin ≥11 mg/kg. In adjusted analysis, daptomycin 8 to <11 mg/kg was associated with higher mortality than daptomycin ≥11 mg/kg [adjusted odds ratio (aOR) 3.11, 95% CI 1.13 to 8.56, *P* = 0.03]. Among isolates with daptomycin MIC = 8 mg/L, 28-day mortality was 54.9% in patients treated with daptomycin 8 to <11 mg/kg versus 29.6% treated with daptomycin ≥11 mg/kg (*P* = 0.04); no benefit was seen for MIC ≥16 mg/L (*P* > 0.99). 28-day mortality was 49.1% (54/110) with daptomycin and 35.0% (7/20) with linezolid (*P* = 0.33); linezolid was not associated with lower mortality than daptomycin in adjusted analysis (*P* = 0.48).

**Conclusions:**

In daptomycin-resistant VRE BSI, daptomycin ≥11 mg/kg was associated with lower 28-day mortality than daptomycin 8 to <11 mg/kg, particularly at MIC = 8 mg/L. Comparisons with the small linezolid arm are exploratory and underpowered. Larger studies are needed to confirm these hypothesis-generating findings.

## Introduction

VRE has emerged as a critical multidrug-resistant pathogen in healthcare settings with growing prevalence worldwide.^1,2^ VRE bloodstream infections (BSIs) are common in immunocompromised and intensive care unit patients and are associated with high mortality.^3,4^ Experts recommend linezolid or high-dose daptomycin (8 to 12 mg/kg) for VRE BSI, and CLSI daptomycin breakpoints for *Enterococcus faecium* are based on daptomycin doses of 8 to 12 mg/kg.^5–7^ Although linezolid has demonstrated favorable outcomes in individual studies and in meta-analyses,^8–10^ its bacteriostatic activity and side effects including thrombocytopenia and lactic acidosis have raised clinical concerns. Daptomycin, a rapid-acting bactericidal agent, has shown superior outcomes in some studies.^11–13^ However, the growing prevalence of daptomycin-resistant VRE threatens the durability of standard therapeutic regimens.^14,15^

Antimicrobial susceptibility testing (AST) results guide treatment decisions for VRE BSI.^16^ Although CLSI has established daptomycin breakpoints for *E. faecium*, current standards stipulate that only BMD results should be used for clinical interpretation.^5,17^ However, manual BMD is labor-intensive, is not universally performed, and is often unavailable within a clinically actionable timeframe.^16,18^ Consequently, most automated AST systems generate daptomycin MICs for *E. faecium* but suppress these values from clinical reports because the methods are not FDA-cleared for this species^18,19^ despite a moderate correlation with BMD values.^20^ Recognition of daptomycin resistance in *E. faecium* therefore depends on reference BMD that is rarely performed in real time, and the optimal definitive therapy for BMD-confirmed daptomycin-resistant VRE BSI, including the choice between linezolid and daptomycin and the optimal daptomycin dosing strategy, remains uncertain.

Within the daptomycin-treated population, prior pharmacokinetic-pharmacodynamic studies and recent observational analyses suggest that higher daptomycin doses may improve outcomes in *E. faecium* BSI, particularly at MICs in the susceptible-dose-dependent (SDD) range.^21–23^ However, the extent to which dose optimization (≥11 mg/kg versus 8 to <11 mg/kg) is associated with improved survival in patients with BMD-confirmed daptomycin-resistant VRE BSI has not been formally estimated using methods that explicitly address confounding by indication, and the comparative outcomes of daptomycin and linezolid in this specific population remain poorly characterized. This study aimed primarily to estimate the dose effect of daptomycin (≥11 mg/kg versus 8 to <11 mg/kg) on 28-day mortality among patients with Sensititre-BMD-confirmed daptomycin-resistant VRE BSI using inverse probability of treatment weighting, and secondarily to describe the clinical characteristics and prognostic factors of this population and the outcomes of patients treated with linezolid as an exploratory comparator.

## Methods

### Patient selection and hospital settings

We conducted a retrospective analysis of a prospectively identified multi-site cohort of patients with VRE BSI managed within the National Taiwan University Hospital (NTUH) system between 22 September 2011 and 11 December 2024. The NTUH system includes the main NTUH campus, a 2200-bed tertiary medical center in Taipei City, the NTUH Yunlin Branch, a 900-bed metropolitan hospital in Yunlin County, and the NTUH Hsinchu Branch, a 600-bed metropolitan hospital in Hsinchu City. Cases were prospectively identified as part of an institutional infection control program and clinical data were subsequently extracted for this study. The Research Ethics Committee of the NTUH (202511027RINB) approved the study protocol and waived the requirement for informed consent.

VRE BSIs were defined as the presence of at least one blood culture positive for VRE. Only the first VRE BSI episode per patient was included during the study period.^22^ Patients were included if they received active definitive therapy for VRE BSI with linezolid or daptomycin (≥8 mg/kg, based on actual body weight). No minimum treatment duration was pre-specified as an inclusion criterion; patients were classified by intention-to-treat according to their initial definitive regimen. For patients receiving daptomycin, the dose (mg/kg) at the initiation of definitive therapy was calculated using the prescribed dose and actual body weight at that time, and was used to categorize patients as 8 to <11 mg/kg or ≥11 mg/kg; subsequent intra-treatment changes in prescribed dose or body weight did not reclassify the patient between groups. Non-hospitalized patients and those younger than 18 years were excluded, as were patients diagnosed with pneumonic BSI. Only episodes caused by vancomycin-resistant *E. faecium* (VREfm) were analyzed; VRE caused by other species were excluded because the CLSI daptomycin breakpoints and the SDD category apply only to *E. faecium*.

### Patient collection and clinical definitions

Baseline characteristics of the enrolled patients were retrieved from their electronic medical records. Primary bacteremia, catheter-related BSIs, immunosuppressant use,^21,24^ and adverse events, including creatine kinase elevation and thrombocytopenia, were defined as previously described.^24^ Immunosuppressant use was defined as the administration of antineoplastic drugs, cyclophosphamide, or other immunosuppressive agents within 6 weeks of bacteremia onset, or the administration of prednisolone at a dosage of ≥20 mg/day for ≥2 weeks or ≥30 mg/day for ≥1 week within 6 weeks of bacteremia onset.^21,24^ The Charlson Comorbidity Index was used to account for underlying health conditions,^25^ and the Pitt Bacteremia Score was used to assess the severity of BSI.^26^

The reference date (day 0) for all outcome assessments was the date the first index blood culture positive for VRE was collected. The primary outcome was 28-day in-hospital mortality. Secondary outcomes included 14-day mortality, microbiological failure, and clinical failure, defined as previously described.^24^ Microbiological failure was defined as either persistent bacteremia (VRE isolated from follow-up blood cultures ≥4 days after BSI onset) or death within 7 days without documented bloodstream clearance. Clinical failure was defined as a composite of 14-day mortality or persistent bacteremia according to a previous study.^24^

### Microbiological studies and AST

During the study period, routine AST in our clinical microbiology laboratory used the VITEK 2 AST (bioMérieux, Marcy-l'Étoile, France); the susceptibility and MIC values of daptomycin for *E. faecium* were not released to treating physicians. *Post hoc* MIC testing was performed using BMD with the Sensititre Gram Positive GPN3F panel (Thermo Fisher Scientific, East Grinstead, UK). The daptomycin two-fold dilution wells on this panel span 0.25 to 8 mg/L with calcium-supplemented Mueller-Hinton broth per CLSI specifications; isolates not inhibited at the highest 8 mg/L well were reported as MIC ≥16 mg/L by the standard two-fold dilution convention. According to the CLSI 2025 breakpoints,^5^ isolates with MIC ≥8 mg/L were classified as resistant and those with MIC ≤4 mg/L as SDD for doses of 8 to 12 mg/kg against *E. faecium.* Linezolid MICs were interpreted according to CLSI criteria.^5^ Because the BMD MICs were generated retrospectively, they were not available at the time of clinical decision-making.

### Statistical analysis

Categorical variables were reported as percentages and compared using two-sided Fisher's exact test. Continuous variables were summarized using medians and interquartile ranges (IQRs) and compared using the Mann-Whitney *U*-test. Statistical significance was defined as a two-sided *P* value of < 0.05.

Univariable analysis of factors associated with 28-day mortality was performed on the full cohort (*n* = 130) for descriptive purposes. Multivariable logistic regression was used for outcome analysis, incorporating variables with *P* ≤ 0.15 from univariable regression; models were constructed using backward stepwise Akaike information criterion (AIC) minimization, and the final model retained only variables with *P* < 0.05. The primary analysis was restricted to the daptomycin-treated subgroup (*n* = 110) and estimated the effect of daptomycin 8 to <11 mg/kg versus ≥11 mg/kg on 28-day mortality. In addition, to address potential imbalance and confounding by indication between treatment groups, augmented inverse probability of treatment weighting (AIPW) was performed within the same daptomycin-treated subgroup. For the AIPW model, propensity-score variables were *a priori* selected^27^ and included Pitt bacteremia score, platelet count, renal replacement therapy, mechanical ventilation, and immunosuppression; the outcome model included treatment group and variables identified as associated with the outcome. Covariate balance after weighting was assessed using standardized mean differences (SMDs). Exploratory analyses including the linezolid arm comprised (i) the same multivariable model applied to the full cohort to compare linezolid with daptomycin, (ii) a separate AIPW for linezolid versus daptomycin, and (iii) a three-arm comparison (linezolid, daptomycin 8 to <11 mg/kg, daptomycin ≥11 mg/kg) using stabilized inverse probability weights with weighted logistic regression. Given the small linezolid arm (*n* = 20), all comparisons involving linezolid were *a priori* underpowered to detect even large differences and are reported for descriptive context only.

Kaplan–Meier curves were used to visualize 28-day in-hospital survival, and Cox proportional-hazards regression was used to estimate adjusted hazard ratios using the same independent predictors of mortality identified in the multivariable logistic regression.

Statistical analyses were performed using Stata software (v.18; StataCorp, College Station, TX, USA).

## Results

### Patient characteristics

**Figure 1.**
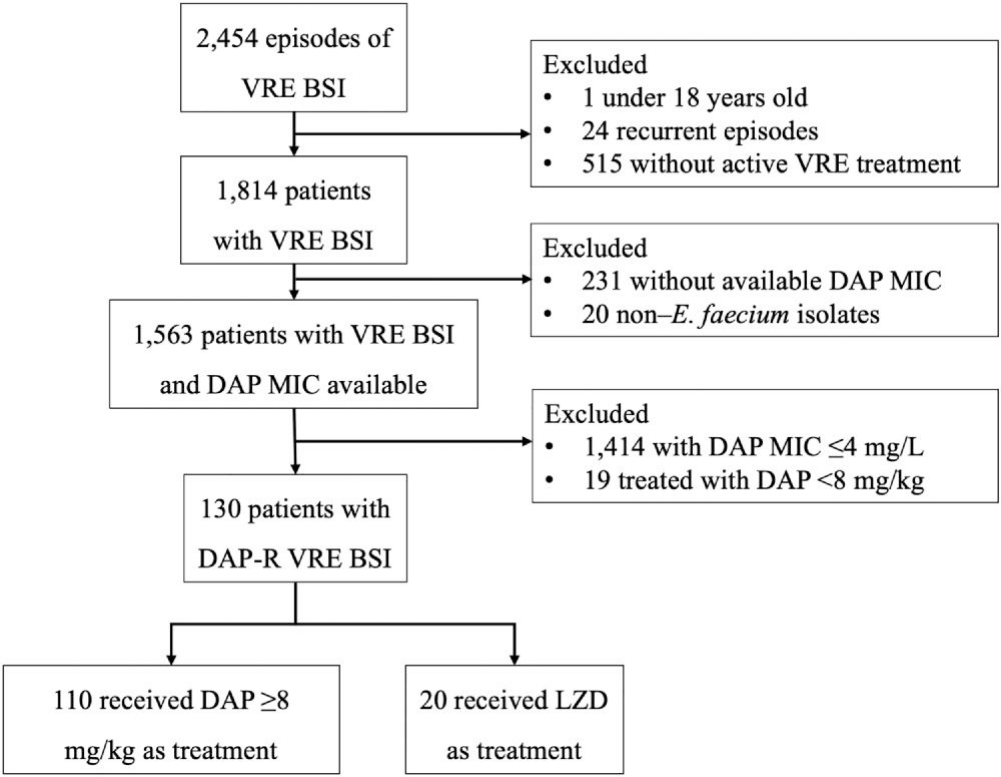
Flow diagram of patient selection for the cohort. BSI, bloodstream infection; DAP, daptomycin; DAP-R, daptomycin-resistant; *E. faecium*, *Enterococcus faecium*; LZD, linezolid.

During the study period, 2454 episodes of VRE BSI were identified (Figure 1). A total of 130 non-duplicate episodes met the criteria for daptomycin resistance (MIC ≥8 mg/L) and were treated with either daptomycin ≥ 8 mg/kg or linezolid; these were included in the analysis.

Demographic and clinical characteristics of the enrolled patients are summarized in Table 1. Their median age was 67.7 years (55.2–78.2), and 78 (60.0%) were male. The median Pitt bacteremia score was 2 [1–4], and the Charlson comorbidity index was 4 [2–5]. VRE BSI developed at a median of 23 days (range, 10–44 days) after hospital admission. Within the preceding 30 days, 82 (63.1%) patients received carbapenems, 59 (45.4%) glycopeptides, and 14 (10.8%) daptomycin.

**Table 1.** Baseline characteristics of patients with BSI caused by daptomycin-resistant vancomycin-resistant *Enterococcus faecium*

	Total (*n* = 130)	Survival (*n* = 69)	Mortality (*n* = 61)	*P* value
Age (years)	67.7 (55.2–78.2)	66.8 (51.3–78.7)	68.6 (57.8–78.1)	0.46
Male	78 (60.0)	44 (63.8)	34 (55.7)	0.38
Body mass index (kg/m^2^)	21.6 (19.1–24.8)	21.6 (18.4–24.0)	21.8 (19.6–25.1)	0.23
Length of hospitalization before VRE BSI onset (days)	23 (10–44)	20 (8–44)	28 (13–42)	0.11
**Underlying conditions**				
Charlson comorbidity index	4 (2–5)	3 (2–5)	4 (2–6)	0.16
Hypertension	64 (49.2)	36 (52.2)	28 (45.9)	0.49
Diabetes mellitus	36 (27.7)	20 (29.0)	16 (26.2)	0.85
Renal replacement therapy^a^	15 (11.5)	11 (15.9)	4 (6.6)	0.11
Solid organ malignancy	27 (20.8)	19 (27.5)	8 (13.1)	0.05
Leukemia	28 (21.5)	11 (15.9)	17 (27.9)	0.13
Lymphoma	13 (10.0)	3 (4.3)	10 (16.4)	0.04
Steroid use	33 (25.4)	12 (17.4)	21 (34.4)	0.03
Chemotherapy	38 (29.2)	18 (26.1)	20 (32.8)	0.44
Immunosuppressants	57 (43.8)	24 (34.8)	33 (54.1)	0.03
Hematopoietic stem cell transplantation	19 (14.6)	7 (10.1)	12 (19.7)	0.14
**Clinical characteristics**				
Neutropenia (ANC < 500/μL)	38 (29.7)	14 (20.3)	24 (40.7)	0.02
Platelet count (×10^4^/μL)	7.0 (2.9–20.7)	15.4 (5.5–26.8)	4.3 (2.2–9.4)	<0.001
Creatinine (mg/dL)	1.2 (0.7–2.3)	1.3 (0.7–2.7)	1.2 (0.7–2.1)	0.66
Pitt bacteremia score	2 (1–4)	2 (0–3)	2 (1–5)	0.01
Polymicrobial infection^b^	39 (30.2)	24 (35.3)	15 (24.6)	0.19
Ventilator use	38 (29.2)	20 (29.0)	18 (29.5)	> 0.99
Intensive care unit stay at VRE BSI onset	35 (26.9)	12 (17.4)	23 (37.7)	0.01
**Infection focus**				
Intra-abdominal infection	13 (10.0)	10 (14.5)	3 (4.9)	0.08
Primary bloodstream infection	61 (46.9)	35 (50.7)	26 (42.6)	0.38
Urinary tract infection	54 (41.5)	24 (34.8)	30 (49.2)	0.11
Daptomycin MIC = 8 mg/L	114 (87.7)	62 (90.0)	52 (85.2)	0.44
Linezolid MIC ≥ 4 mg/L	15 (11.5)	8 (11.6)	7 (11.5)	> 0.99
Carbapenem use within 30 days before VRE BSI onset	82 (63.1)	43 (62.3)	39 (63.9)	0.86
Glycopeptides use within 30 days before VRE BSI onset	59 (45.4)	26 (37.7)	33 (54.1)	0.08
**Treatment**				
Time from VRE BSI onset to active VRE treatment (days)	2 (1–3)	2 (1–3)	2 (1–2)	0.07
Daptomycin dose (mg/kg)	10.2 (9.4–11.1)	10.2 (9.7–11.8)	10.0 (9.3–10.8)	0.06
Linezolid treatment	20 (15.4)	13 (18.8)	7 (11.5)	0.33
Combination antibiotic therapy^c^	96 (73.8)	50 (72.5)	46 (75.4)	0.70

Data are presented as medians (interquartile range) or number (percentage).

ANC, absolute neutrophil count; BSI, bloodstream infection.

^a^Renal replacement therapy includes hemodialysis and peritoneal dialysis.

^b^Polymicrobial infection is defined as concurrent bloodstream infection with another pathogen at the time of VRE BSI.

^c^Combination antibiotic therapy is defined as any beta-lactam or fosfomycin use during active VRE treatment.

### Univariate analysis of factors associated with 28-day mortality

The factors associated with 28-day mortality are summarized in Table 1. Compared with survivors, patients in the mortality group had higher Pitt bacteremia scores and lower platelet counts, consistent with greater acute illness severity. The median time from BSI onset to targeted VRE treatment was 2 days in the mortality and survival groups (*P* = 0.07). The 28-day mortality rate in the daptomycin group was 49.1% (54/110), which was higher than that in the linezolid cohort (35.0%, 7/20), although the difference was not statistically significant (*P* = 0.33). The median daptomycin dose was 10.0 mg/kg in both the mortality group and 10.2 mg/kg in the survival group (*P* = 0.06). Among patients receiving daptomycin, the mortality rate was 34.4% (11/32) for those receiving ≥11 mg/kg and 55.1% (43/78) for those receiving 8 to <11 mg/kg (*P* = 0.06). When stratified by daptomycin MIC, the 28-day mortality rate was 45.6% (52/114) in patients with an MIC of 8 mg/L and 56.3% (9/16) in those with an MIC of ≥ 16 mg/L (*P* = 0.44).

### Multivariable logistic regression analysis of factors associated with 28-day mortality

**Figure 2.**
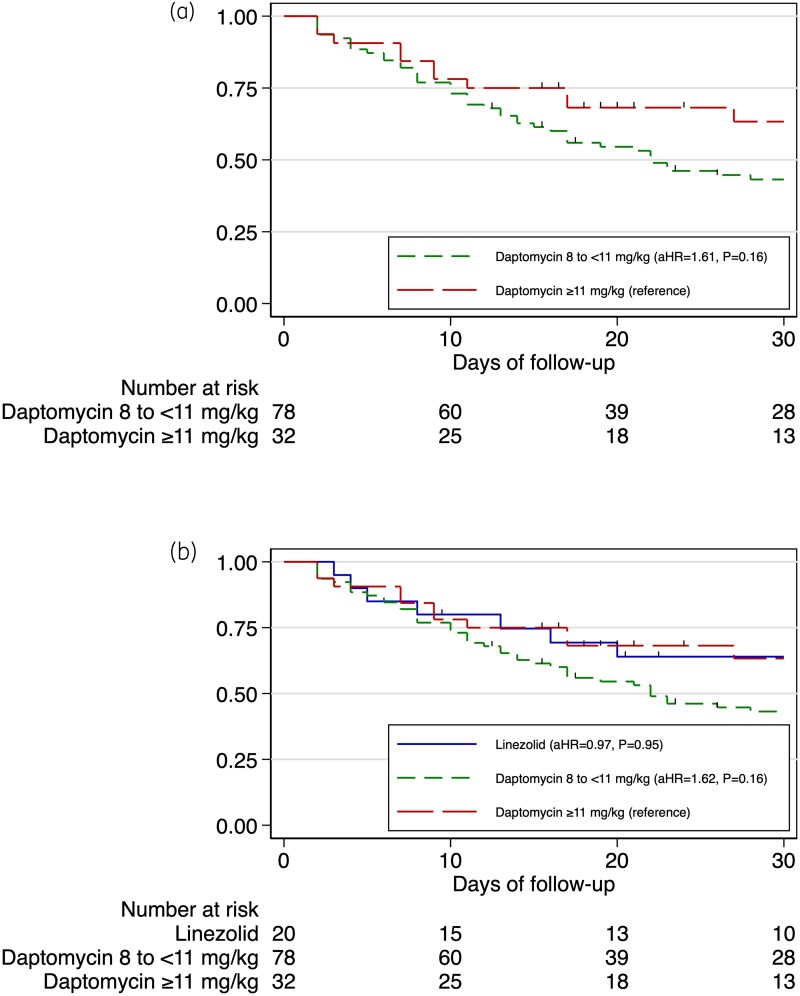
Kaplan–Meier 28-day in-hospital survival curves. (a) Primary analysis restricted to the daptomycin-treated subgroup (*n* = 110), comparing daptomycin 8 to <11 mg/kg versus ≥11 mg/kg. Adjusted hazard ratio for daptomycin 8 to <11 mg/kg versus ≥11 mg/kg: aHR 1.61 (95% CI 0.83 to 3.14, *P* = 0.16). (b) Exploratory three-arm analysis on the full cohort (*n* = 130), with daptomycin ≥11 mg/kg as the reference. Adjusted hazard ratios: linezolid 0.97 (95% CI 0.37 to 2.53, *P* = 0.95); daptomycin 8 to <11 mg/kg 1.62 (95% CI 0.83 to 3.14, *P* = 0.16). All Cox proportional-hazards models were adjusted for Pitt bacteremia score, platelet count, and renal replacement therapy.

In the primary multivariable logistic regression, restricted to the daptomycin-treated subgroup (*n* = 110), daptomycin 8 to <11 mg/kg was associated with significantly higher 28-day mortality than daptomycin ≥11 mg/kg after adjustment for the independent predictors of mortality (Pitt bacteremia score, platelet count, and renal replacement therapy) (Table 2; adjusted odds ratio [aOR] 3.11, 95% CI 1.13 to 8.56, *P* = 0.03). The corresponding Kaplan–Meier survival curves for the two daptomycin dose groups are shown in Figure 2A.

**Table 2.** Primary multivariable logistic regression for 28-day mortality, restricted to daptomycin-treated patients (*n* = 110)

Variable	aOR	95% CI	*P* value
Pitt bacteremia score (per 1-point increase)	1.26	1.02 to 1.56	0.03
Platelet count (per 10^4^/μL)	0.89	0.85 to 0.94	<0.001
Renal replacement therapy^a^	0.18	0.04 to 0.83	0.03
Daptomycin 8 to <11 versus ≥11 mg/kg (reference)	3.11	1.13 to 8.56	0.03

aOR, adjusted odds ratio; CI, confidence interval.

^a^Renal replacement therapy includes hemodialysis and peritoneal dialysis.

### Augmented inverse probability weighting analysis of daptomycin dose effect

To complement the primary multivariable result, we estimated the average treatment effect of daptomycin ≥11 mg/kg versus 8 to <11 mg/kg on 28-day mortality within the same daptomycin-treated subgroup (*n* = 110) using AIPW. Baseline characteristics and covariate balance before and after AIPW for this primary dose comparison are summarized in Table S1 (available as Supplementary data at *JAC-AMR* Online); all five propensity-score variables achieved |SMD| < 0.05 after weighting, while residual imbalance for several non-PS-model variables is shown in the same table. The AIPW-estimated 28-day mortality was 35.1% in the daptomycin ≥11 mg/kg group versus 54.9% in the daptomycin 8 to <11 mg/kg group (ATE −0.198, 95% CI −0.37 to −0.03; bootstrap *P* = 0.02). The estimate was directionally and statistically consistent with the primary multivariable result above.

Of the 110 patients in the primary analysis, 1 (0.9%) received only a single day of definitive therapy (daptomycin 8 to <11 mg/kg) and 6 (5.5%) had a treatment switch during definitive therapy (all from daptomycin to linezolid; median time to switch 4 days, IQR 3 to 5). Because BMD MIC results were not available to treating physicians, switches reflected clinical considerations rather than knowledge of phenotypic daptomycin resistance. Sensitivity analyses excluding the one short-duration patient (*n* = 109) or the six switchers (*n* = 104) did not materially change the primary AIPW result (ATE = −0.20, 95% CI −0.37 to −0.02, *P* = 0.03; and ATE = −0.21, 95% CI −0.39 to −0.04, *P* = 0.01, respectively).

### Interaction analysis between daptomycin MIC and treatment on outcomes

**Figure 3.**
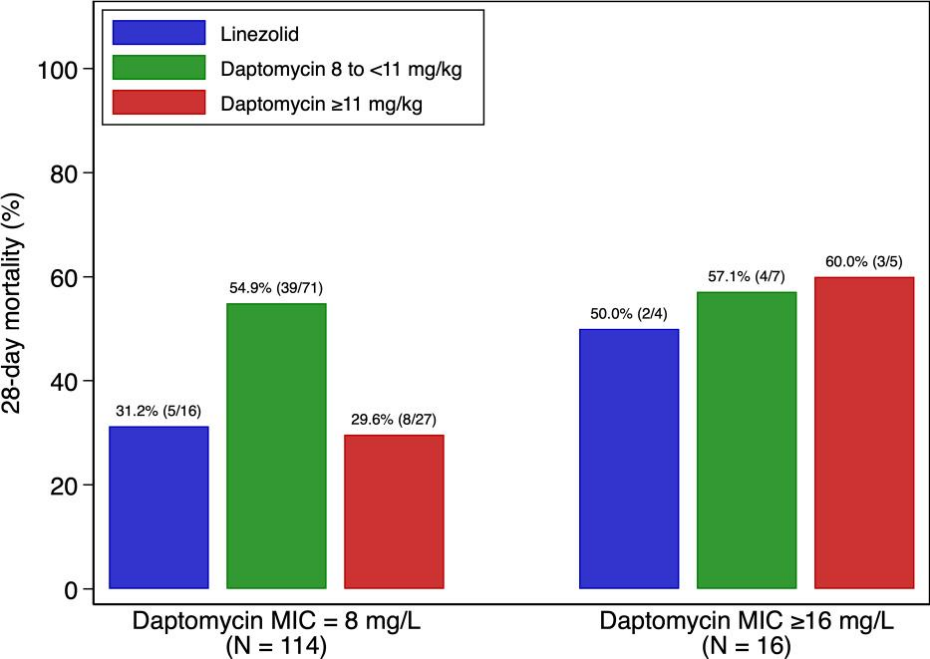
28-day mortality by treatment group and daptomycin MIC. Bars show 28-day in-hospital mortality by treatment arm (linezolid, daptomycin 8 to <11 mg/kg, daptomycin ≥11 mg/kg), stratified by daptomycin MIC (= 8 mg/L versus ≥16 mg/L). Numbers above each bar are the mortality percentage and (deaths/*n* at risk).

When outcomes were examined in relation to daptomycin MIC and treatment choice, mortality was numerically higher in the daptomycin group than in the linezolid group at both MIC 8 mg/L (48.0% versus 31.3%; *P* = 0.28) and MIC ≥16 mg/L (58.3% versus 50.0%; *P* > 0.99), although these differences were not statistically significant (Table 3). We then compared linezolid with different daptomycin doses stratified by MIC. For isolates with daptomycin MIC ≥16 mg/L, 28-day mortality was 50.0% (2/4) in the linezolid group, 57.1% (4/7) in the daptomycin 8 to <11 mg/kg group, and 60.0% (3/5) in the daptomycin ≥11 mg/kg group (*P* > 0.99). On the other hand, for isolates with MIC = 8 mg/L, 28-day mortality was 31.3% (5/16) with linezolid, 54.9% (39/71) with daptomycin 8 to <11 mg/kg, and 29.6% (8/27) with daptomycin ≥11 mg/kg (*P* = 0.04) (Table 3, Figure 3). Marginal analysis adjusted for renal replacement therapy, Pitt bacteremia score, and platelet count confirmed that daptomycin ≥11 mg/kg reduced 28-day mortality by 20.2% (absolute risk reduction) compared with daptomycin 8 to <11 mg/kg when MIC = 8 mg/L (*P* = 0.03), but not when MIC ≥16 mg/L (*P* = 0.42).

**Table 3.** 28-day mortality by treatment, daptomycin dose, and minimum inhibitory concentration strata

Daptomycin MIC	Treatment	*n*/*N* (%) Mortality	*P* value^a^
**Linezolid versus all Daptomycin**			
8 mg/L (*n* = 114)	Linezolid	5/16 (31.3)	
	Daptomycin (all doses)	47/98 (48.0)	0.28
≥16 mg/L (*n* = 16)	Linezolid	2/4 (50.0)	
	Daptomycin (all doses)	7/12 (58.3)	> 0.99
			
**Linezolid versus Daptomycin stratified by dose**			
8 mg/L (*n* = 114)	Linezolid	5/16 (31.3)	
	Daptomycin 8 to <11 mg/kg	39/71 (54.9)	
	Daptomycin ≥11 mg/kg	8/27 (29.6)	0.04
≥16 mg/L (*n* = 16)	Linezolid	2/4 (50.0)	
	Daptomycin 8 to <11 mg/kg	4/7 (57.1)	
	Daptomycin ≥11 mg/kg	3/5 (60.0)	> 0.99

^a^Two-sided Fisher’s exact test comparing treatments within each MIC stratum. The upper section compares linezolid with all daptomycin regimens, and the lower section compares linezolid with daptomycin stratified by dose.

### Secondary outcome analysis

Regarding secondary outcomes, there were no significant differences between patients treated with daptomycin and those treated with linezolid in terms of the rates of persistent infection (25.5% versus 25.4%, *P* > 0.99), 14-day mortality (33.6% versus 25.0%, *P* = 0.61), microbiological failure (30.9% versus 25.0%, *P* = 0.79), or clinical failure (40.9% versus 35.0%, *P* = 0.81). There was no significant difference in elevated creatinine kinase levels (16.2% versus 28.6%, *P* = 0.60) or thrombocytopenia (57.3% versus 42.1%, *P* = 0.32) between patients receiving daptomycin and linezolid.

### Exploratory analyses including the linezolid arm

We performed an exploratory multivariable logistic regression on the full cohort (*n* = 130). In a binary comparison (Model 1, Table 4), linezolid was not associated with lower mortality than daptomycin (aOR 0.64, 95% CI 0.19 to 2.16, *P* = 0.48). With a three-level treatment variable (linezolid, daptomycin 8 to <11 mg/kg, daptomycin ≥11 mg/kg) and daptomycin ≥11 mg/kg as the reference category (Table 4, Model 2), the dose contrast was directionally and statistically consistent with the primary daptomycin-only model in Table 2 (daptomycin 8 to <11 versus ≥11 mg/kg: aOR 3.02, 95% CI 1.12 to 8.17, *P* = 0.03), and linezolid did not differ significantly from daptomycin ≥11 mg/kg (aOR 1.39, 95% CI 0.34 to 5.75, *P* = 0.64). Baseline characteristics and covariate balance before and after AIPW for the daptomycin-versus-linezolid comparison are summarized in Table S2. A separate exploratory AIPW analysis (binary linezolid versus daptomycin) was conducted; the average treatment effect (daptomycin minus linezolid) on 28-day mortality was not statistically significant (weighted mortality 47.0% in the daptomycin arm versus 51.8% in the linezolid arm; ATE −0.048, *P* = 0.72). In a three-arm stabilized IPW (inverse probability weighting) analysis, the weighted 28-day mortality was 30.5% in the daptomycin ≥11 mg/kg arm, 54.3% in the daptomycin 8 to <11 mg/kg arm, and 60.9% in the linezolid arm. The corresponding three-arm Kaplan–Meier survival curves are shown in Figure 2B. Of note, however, covariate balance after weighting in this linezolid versus daptomycin comparison remained substantially imbalanced for several baseline characteristics (Table S2), reflecting the small size of the linezolid arm; the linezolid results should therefore be regarded only as exploratory and hypothesis-generating, not as evidence of either equivalence or difference.

**Table 4.** Exploratory multivariable logistic regression for 28-day mortality, full cohort (*n* = 130)

Variable	aOR	95% CI	*P* value
**Model 1: linezolid versus daptomycin**			
Pitt bacteremia score (per 1-point increase)	1.25	1.05 to 1.50	0.01
Platelet count (per 10^4^/μL)	0.90	0.86 to 0.95	<0.001
Renal replacement therapy^a^	0.24	0.06 to 0.90	0.03
Linezolid versus daptomycin (reference)	0.64	0.19 to 2.16	0.48
**Model 2: dose-stratified comparison**			
Pitt bacteremia score (per 1-point increase)	1.28	1.06 to 1.54	0.01
Platelet count (per 10^4^/μL)	0.90	0.86 to 0.94	<0.001
Renal replacement therapy^a^	0.23	0.06 to 0.87	0.03
Linezolid versus daptomycin ≥11 mg/kg reference)	1.39	0.34 to 5.75	0.64
Daptomycin 8 to <11 versus ≥11 mg/kg (reference)	3.02	1.12 to 8.17	0.03

aOR, adjusted odds ratio; CI, confidence interval.

^a^Renal replacement therapy includes hemodialysis and peritoneal dialysis.

## Discussion

In this study, we characterized the clinical features and outcomes of patients with BSI caused by daptomycin-resistant VRE faecium and explored the impact of daptomycin dosing strategy. Several observations emerged from this analysis. First, in the primary multivariable logistic regression restricted to the daptomycin-treated subgroup (Table 2), higher Pitt bacteremia score and lower platelet count were independently associated with 28-day mortality, consistent with the recognized influence of acute illness severity and host reserves on outcomes in VRE BSI.^11,28^ The association between renal replacement therapy and lower mortality should be interpreted with caution and may reflect residual confounding (for example, differences in case selection or intensity of monitoring) rather than a causal protective effect; this finding warrants exploration in larger cohorts. Second, the dose-response signal within the daptomycin arm was concordant across two complementary analytic strategies. The primary multivariable logistic regression showed that daptomycin 8 to <11 mg/kg was associated with higher 28-day mortality than daptomycin ≥11 mg/kg (Table 2; aOR 3.11, 95% CI 1.13 to 8.56, *P* = 0.03). The primary AIPW analysis on the same subgroup, with explicit covariate balance reporting (Table S1), was directionally and statistically consistent (ATE −0.198, 95% CI −0.37 to −0.03, *P* = 0.02). The convergence across methods strengthens the inference that dose optimization is associated with improved survival in this population. The dose effect appeared most pronounced for isolates with daptomycin MIC = 8 mg/L (*n* = 27 with ≥11 mg/kg) and should still be regarded as exploratory and hypothesis-generating given the modest sample size of the high-dose subgroup. Third, comparisons with the linezolid arm (*n* = 20) are reported for descriptive context only and are insufficient to support conclusions regarding relative effectiveness.

In our exploratory comparison of linezolid versus daptomycin, we did not detect a difference in 28-day mortality between the two arms. This null result should not be interpreted as evidence of equivalence and should not be used to diminish the clinical importance of AST. Several factors limit inference about the relative effectiveness of these regimens in this setting. First, the sample size was relatively small, particularly in the linezolid arm (*n* = 20), which *a priori* limited the statistical power to detect even large differences. Second, daptomycin MIC testing is poorly reproducible, with values varying by method and inoculum, and exhibiting substantial inter-laboratory variability.^29–31^ The regulatory uncertainty surrounding daptomycin breakpoints reflects these issues; although the FDA recently recognized CLSI SDD breakpoints for daptomycin against *E. faecium,*^19^ EUCAST has not established breakpoints, citing concerns regarding MIC reproducibility and pharmacodynamic target attainment.^32^ Third, although we used Sensititre, an FDA-approved commercial BMD platform,^33^ it is not identical to manual BMD, and the clinical significance of the minor MIC differences between the methods remains uncertain. Finally, the current daptomycin MIC testing may not fully reflect treatment failure *in vivo.*^34^

In a recent study, high-dose daptomycin (8 to 12 mg/kg) was associated with encouraging short-term survival in *E. faecium* BSI with daptomycin MICs in the CLSI SDD range,^23^ suggesting that high-dose therapy can be effective for isolates in the SDD range. Our cohort of BMD-confirmed daptomycin-resistant VREfm strains extends this evidence to isolates at the upper end of the MIC distribution. We examined two MIC strata, 8 mg/L (*n* = 114, 87.7%) and ≥ 16 mg/L (*n* = 16, 12.3%). Despite the known pharmacodynamic relationship between *f*AUC/MIC and efficacy,^20,35,36^ the 28-day mortality did not differ significantly between these MIC groups among daptomycin-treated patients (48.0% [47/98] versus 58.3% [7/12], *P* = 0.55). Although one study reported an association between an elevated daptomycin MIC and microbiological failure,^37^ most clinical studies have not demonstrated a consistent correlation between higher MICs and worse outcomes.^24,28,38^ This likely reflects both MIC variability and the complex interplay between host factors, antibiotic exposure, and pathogen dynamics in VRE BSI. Notably, the survival benefit of daptomycin ≥11 mg/kg compared to 8 to <11 mg/kg was observed primarily in isolates with MIC = 8 mg/L (29.6% versus 54.9% mortality, *P* = 0.04) but not in those with MIC ≥16 mg/L (60.0% versus 57.1%, *P* > 0.99). However, the small sample size in the higher MIC stratum (*n* = 16) substantially limits inferences of dose responses at these resistance levels and warrants confirmation in larger cohorts.

Our study had certain limitations. First, the sample size, especially in the linezolid arm (*n* = 20), was limited, which likely reduced the statistical power of the subgroup analyses and detected modest treatment differences. Second, manual BMD was not used, which precludes a definitive classification of MICs. However, using an FDA-cleared commercial BMD platform (Sensititre) improved the practical relevance of our findings to routine clinical practice. Third, the observational design introduced the possibility of unmeasured confounding factors. Treatment was not randomized and confounding by indication cannot be excluded. Although we applied AIPW in addition to multivariable logistic regression, the covariate balance remained imperfect, reflecting the limited sample size. Fourth, we did not evaluate the underlying mechanisms of resistance. Although all isolates were phenotypically resistant to daptomycin, we did not confirm the presence of known resistance genes (e.g. mutations in *liaFSR*, *liaX*, or *cls*),^29,39^ which may have led to misclassification bias. Genotypic confirmation would help to more definitively categorize daptomycin resistance and distinguish true resistance from testing variability. We acknowledge that our composite definitions of microbiological and clinical failure capture survival and bacteremia clearance but do not directly measure symptomatic clinical improvement; future studies in this population should consider standardized organ-failure endpoints. In the exploratory analyses involving the linezolid arm, despite the application of augmented IPW, covariate balance after weighting remained substantially imbalanced for several baseline characteristics (Table S2), reflecting the small size of the linezolid arm (*n* = 20). Although AIPW is doubly robust (consistent if either the propensity-score or the outcome model is correctly specified), residual confounding by variables not included in the propensity-score model cannot be excluded; the linezolid comparison should therefore be regarded only as exploratory and hypothesis-generating.

In conclusion, in this single-center retrospective cohort of 130 patients with BSI caused by Sensititre-BMD-confirmed daptomycin-resistant VRE faecium, daptomycin ≥11 mg/kg was associated with lower 28-day mortality than daptomycin 8 to <11 mg/kg in both the multivariable logistic regression (aOR 3.11, 95% CI 1.13 to 8.56, *P* = 0.03) and the primary AIPW analysis (ATE −0.198, 95% CI −0.37 to −0.03, *P* = 0.02), particularly among isolates with MIC = 8 mg/L. Comparisons with the small linezolid arm (*n* = 20) are descriptive and underpowered, and these findings should be interpreted as exploratory and hypothesis-generating. Our analysis was restricted to isolates classified as daptomycin-resistant by Sensititre-BMD and did not assess outcomes across the full spectrum of automated AST results or directly evaluate the impact of suppressing those results. Larger, multicenter studies that explicitly link automated AST results, BMD MICs, dosing strategies and molecular resistance mechanisms with patient outcomes are required to define the optimal management of daptomycin-resistant VRE BSI and to clarify how daptomycin MICs should be incorporated into reporting and dosing decisions.

## Supplementary Material

dlag108_Supplementary_Data

## Supplementary data

Tables S1 and S2 are available as Supplementary data at *JAC-AMR* Online.
