# Supplementary material for: Treatment outcomes of daptomycin- and vancomycin-resistant Enterococcus faecium bloodstream infection
Source: JAC Antimicrob Resist. 2026 Jun 12;8(3):dlag108. doi: 10.1093/jacamr/dlag108 (PMC13260658; doi:10.1093/jacamr/dlag108)
Supplement: dlag108_Supplementary_Data [file dlag108_supplementary_data.docx]

**Supplementary data: Treatment outcomes of daptomycin and vancomycin-resistant *Enterococcus faecium* bloodstream infection**

Wei-Ting LIN^1^, Jia-Ling YANG ^1^, Chi-Ying LIN ^2^, Sung-Hsi HUANG ^3^, Yu-Chung CHUANG^1, *^, Jann-Tay WANG ^1^, Yee-Chun CHEN ^1^, Shan-Chwen CHANG ^1^

^1^Department of Internal Medicine, National Taiwan University Hospital, Taipei City 100225, Taiwan;

^2^Department of Internal Medicine, National Taiwan University Hospital Yun-Lin Branch, Yun-Lin County 640203, Taiwan;

^3^Department of Internal Medicine, National Taiwan University Hospital Hsin-Chu Branch, Hsin-Chu City 300195, Taiwan.

**Running title:** Treating Daptomycin-Resistant VRE BSI

**Word counts:** synopsis – 250 (limits: 250),

*** Corresponding author**

Yu-Chung Chuang, M.D., PhD,
Professor, Dept. Internal Medicine, National Taiwan University Hospital,
7, Zhongshan S. Rd., Zhongzheng Dist., Taipei City 100225, Taiwan
Email: [weischuang@gmail.com](file:///Users/weischuang/Downloads/weischuang@gmail.com)
Telephone: +886-2-2312-3456. Ext 265959

**Table of Contents**

Supplementary Table S1……………………………………………………………….3

Supplementary Table S2……………………………………………….………………7

**Supplementary Table S1.** **Baseline characteristics and covariate balance for the primary dose comparison: daptomycin 8 to <11 mg/kg versus daptomycin ≥11 mg/kg.**

|  | **Daptomycin 8 to <11 mg/kg n=78** | **Daptomycin** ≥**11 mg/kg n=32** | ***P* value** | **Raw SMD** | **Weighted SMD** | **PS model** |
| --- | --- | --- | --- | --- | --- | --- |
| **Demographics** | | | | | | |
| Age (years) | 67.7 (55.0-80.6) | 65.4 (55.1-77.0) | 0.54 | -0.136 | -0.206 |  |
| Male | 47 (60.3) | 19 (59.4) | > 0.99 | -0.018 | -0.090 |  |
| Body mass index (kg/m²) | 22.3 (19.4-24.9) | 20.2 (18.3-23.8) | 0.08 | -0.345 | -0.320 |  |
| Length of hospitalization before VRE BSI onset (days) | 24.0 (12.0-42.0) | 23.0 (7.5-64.5) | 0.90 | 0.249 | 0.274 |  |
| **Underlying conditions** | | | | | | |
| Charlson comorbidity index | 4.0 (2.0-5.0) | 3.0 (2.0-6.0) | 0.97 | -0.017 | 0.030 |  |
| Hypertension | 40 (51.3) | 13 (40.6) | 0.40 | -0.213 | -0.268 |  |
| Diabetes mellitus | 20 (25.6) | 12 (37.5) | 0.25 | 0.254 | 0.199 |  |
| Renal replacement therapy | 9 (11.5) | 3 (9.4) | > 0.99 | -0.070 | -0.033 | Yes |
| Solid organ malignancy | 13 (16.7) | 10 (31.2) | 0.12 | 0.343 | 0.306 |  |
| Leukemia | 19 (24.4) | 7 (21.9) | > 0.99 | -0.058 | 0.043 |  |
| Lymphoma | 12 (15.4) | 1 (3.1) | 0.10 | -0.429 | -0.414 |  |
| Steroid use | 24 (30.8) | 5 (15.6) | 0.15 | -0.361 | -0.306 |  |
| Chemotherapy | 23 (29.5) | 13 (40.6) | 0.27 | 0.232 | 0.406 |  |
| Immunosuppressants | 39 (50.0) | 13 (40.6) | 0.41 | -0.187 | 0.007 | Yes |
| Hematopoietic stem cell transplantation | 15 (19.2) | 3 (9.4) | 0.26 | -0.282 | -0.239 |  |
| **Clinical characteristics** | | | | | | |
| Neutropenia (ANC < 500/μL) | 25 (32.9) | 11 (34.4) | > 0.99 | 0.031 | 0.170 |  |
| Platelet count (×10⁴/μL) | 6.0 (2.9-19.6) | 7.1 (2.5-17.8) | 0.81 | 0.182 | -0.034 | Yes |
| Creatinine (mg/dL) | 1.3 (0.8-2.8) | 0.9 (0.6-1.5) | 0.03 | -0.560 | -0.544 |  |
| Pitt bacteremia score | 2.0 (1.0-4.0) | 2.0 (1.0-4.0) | 0.72 | 0.050 | 0.019 | Yes |
| Ventilator use | 18 (23.1) | 8 (25.0) | 0.81 | 0.045 | 0.000 | Yes |
| Intensive care unit stay at VRE BSI onset | 20 (25.6) | 7 (21.9) | 0.81 | -0.088 | -0.101 |  |
| Daptomycin MIC = 8 mg/L | 71 (91.0) | 27 (84.4) | 0.33 | -0.201 | -0.249 |  |
| Linezolid MIC ≥ 4 mg/L | 9 (11.5) | 3 (9.4) | > 0.99 | -0.070 | -0.026 |  |
| Carbapenem use within 30 days before VRE BSI onset | 53 (67.9) | 19 (59.4) | 0.51 | -0.177 | -0.127 |  |
| Glycopeptides use within 30 days before VRE BSI onset | 37 (47.4) | 15 (46.9) | > 0.99 | -0.011 | 0.040 |  |
| Daptomycin use within 30 days before VRE BSI onset | 8 (10.3) | 4 (12.5) | 0.74 | 0.070 | 0.091 |  |
| Time to antibiotic administration | 2.0 (1.0-3.0) | 2.0 (1.0-3.0) | 0.77 | -0.085 | -0.113 |  |
| **Infection focus** | | | | | | |
| Intraabdominal infection | 4 (5.1) | 6 (18.8) | 0.06 | 0.424 | 0.411 |  |
| Surgical wound infection | 3 (3.8) | 2 (6.2) | 0.63 | 0.109 | 0.070 |  |
| Catheter related blood stream infection | 2 (2.6) | 1 (3.1) | > 0.99 | 0.033 | -0.003 |  |
| Primary bloodstream infection | 38 (48.7) | 12 (37.5) | 0.30 | -0.225 | -0.238 |  |
| Urinary tract infection | 33 (42.3) | 14 (43.8) | > 0.99 | 0.029 | 0.063 |  |
| **Treatment and outcomes** | | | | | | |
| Persistent infection | 19 (24.4) | 9 (28.1) | 0.81 |  |  |  |
| 14-Day mortality | 29 (37.2) | 8 (25.0) | 0.27 |  |  |  |
| Microbiological failure | 26 (33.3) | 8 (25.0) | 0.50 |  |  |  |
| Clinical failure | 33 (42.3) | 12 (37.5) | 0.68 |  |  |  |
| 28-Day mortality | 43 (55.1) | 11 (34.4) | 0.06 |  |  |  |

Values are presented as median (IQR) or n (%). *P* values compare patients receiving daptomycin 8 to <11 mg/kg with those receiving daptomycin ≥11 mg/kg using the Wilcoxon rank-sum test for continuous variables and Fisher's exact test for categorical variables. Raw and weighted standardized mean differences (SMDs) are shown only for baseline/pre-treatment covariates and treatment-timing variables. SMDs were not calculated for post-baseline outcome rows. Variables marked Yes in the PS model column were included in the propensity-score model used for augmented inverse probability weighting.

Abbreviations: ANC, absolute neutrophil count; BSI, bloodstream infection; IQR, interquartile range; PS, propensity score; SMD, standardized mean difference.

**Supplementary Table S2. Baseline characteristics and covariate balance for the exploratory comparison of daptomycin versus linezolid.**

|  | **Daptomycin n=110** | **Linezolid n=20** | ***P* value** | **Raw SMD** | **Weighted SMD** | **PS model** |
| --- | --- | --- | --- | --- | --- | --- |
| **Demographics** | | | | | | |
| Age (years) | 67.3 (55.0-78.8) | 69.2 (57.4-77.6) | 0.73 | 0.129 | -0.140 |  |
| Male | 66 (60.0) | 12 (60.0) | > 0.99 | 0.000 | -0.483 |  |
| Body mass index (kg/m²) | 21.6 (19.1-24.8) | 21.2 (18.9-23.8) | 0.62 | -0.192 | -0.439 |  |
| Length of hospitalization before VRE BSI onset (days) | 23.0 (11.0-45.0) | 17.5 (8.5-37.0) | 0.34 | 0.027 | -0.103 |  |
| **Underlying conditions** | | | | | | |
| Charlson comorbidity index | 4.0 (2.0-5.0) | 2.5 (2.0-4.5) | 0.12 | -0.347 | -0.222 |  |
| Hypertension | 53 (48.2) | 11 (55.0) | 0.63 | 0.135 | -0.354 |  |
| Diabetes mellitus | 32 (29.1) | 4 (20.0) | 0.59 | -0.210 | -0.355 |  |
| Renal replacement therapy | 12 (10.9) | 3 (15.0) | 0.70 | 0.120 | -0.191 | Yes |
| Solid organ malignancy | 23 (20.9) | 4 (20.0) | > 0.99 | -0.022 | -0.299 |  |
| Leukemia | 26 (23.6) | 2 (10.0) | 0.24 | -0.366 | 0.281 |  |
| Lymphoma | 13 (11.8) | 0 (0.0) | 0.22 | -0.515 | -0.491 |  |
| Steroid use | 29 (26.4) | 4 (20.0) | 0.78 | -0.149 | -0.329 |  |
| Chemotherapy | 36 (32.7) | 2 (10.0) | 0.06 | -0.571 | 0.333 |  |
| Immunosuppressants | 52 (47.3) | 5 (25.0) | 0.09 | -0.470 | 0.245 | Yes |
| Hematopoietic stem cell transplantation | 18 (16.4) | 1 (5.0) | 0.30 | -0.371 | -0.392 |  |
| **Clinical characteristics** | | | | | | |
| Neutropenia (ANC < 500/μL) | 36 (33.3) | 2 (10.0) | 0.04 | -0.584 | 0.078 |  |
| Platelet count (×10⁴/μL) | 6.4 (2.8-19.6) | 15.3 (5.5-25.0) | 0.06 | 0.407 | -0.015 | Yes |
| Creatinine (mg/dL) | 1.2 (0.7-2.4) | 1.4 (0.7-1.9) | 0.70 | 0.093 | -0.101 |  |
| Pitt bacteremia score | 2.0 (1.0-4.0) | 2.5 (1.5-5.5) | 0.13 | 0.359 | 0.464 | Yes |
| Ventilator use | 26 (23.6) | 12 (60.0) | 0.002 | 0.780 | -0.062 | Yes |
| Intensive care unit stay at VRE BSI onset | 27 (24.5) | 8 (40.0) | 0.17 | 0.330 | -0.112 |  |
| Daptomycin MIC = 8 mg/L | 98 (89.1) | 16 (80.0) | 0.27 | -0.249 | -0.727 |  |
| Linezolid MIC ≥ 4 mg/L | 12 (10.9) | 0 (0.0) | 0.21 | -0.493 | -0.463 |  |
| Carbapenem use within 30 days before VRE BSI onset | 72 (65.5) | 10 (50.0) | 0.21 | -0.312 | 0.164 |  |
| Glycopeptides use within 30 days before VRE BSI onset | 52 (47.3) | 7 (35.0) | 0.34 | -0.248 | 0.023 |  |
| Daptomycin use within 30 days before VRE BSI onset | 12 (10.9) | 2 (10.0) | > 0.99 | -0.029 | -0.204 |  |
| Time to antibiotic administration | 2.0 (1.0-3.0) | 2.0 (1.5-3.0) | 0.42 | -0.070 | -0.265 |  |
| **Infection focus** | | | | | | |
| Intraabdominal infection | 10 (9.1) | 3 (15.0) | 0.42 | 0.179 | -0.164 |  |
| Surgical wound infection | 5 (4.5) | 1 (5.0) | > 0.99 | 0.021 | -0.136 |  |
| Catheter related blood stream infection | 3 (2.7) | 1 (5.0) | 0.49 | 0.116 | 0.450 |  |
| Primary bloodstream infection | 50 (45.5) | 11 (55.0) | 0.47 | 0.189 | -0.308 |  |
| Urinary tract infection | 47 (42.7) | 7 (35.0) | 0.63 | -0.157 | 0.215 |  |
| **Treatment and outcomes** | | | | | | |
| Persistent infection | 28 (25.5) | 5 (25.0) | > 0.99 |  |  |  |
| 14-Day mortality | 37 (33.6) | 5 (25.0) | 0.60 |  |  |  |
| Microbiological failure | 34 (30.9) | 5 (25.0) | 0.79 |  |  |  |
| Clinical failure | 45 (40.9) | 7 (35.0) | 0.80 |  |  |  |
| 28-Day mortality | 54 (49.1) | 7 (35.0) | 0.33 |  |  |  |

Values are presented as median (IQR) or n (%). P values compare patients receiving daptomycin with those receiving linezolid using the Wilcoxon rank-sum test for continuous variables and Fisher's exact test for categorical variables. Raw and weighted standardized mean differences (SMDs) are shown only for baseline/pre-treatment covariates and treatment-timing variables. SMDs were calculated as linezolid minus daptomycin and were not calculated for post-baseline outcome rows. Variables marked Yes in the PS model column were included in the propensity-score model used for augmented inverse probability weighting. Because the linezolid group was small, residual imbalance after weighting should be interpreted cautiously.

Abbreviations: ANC, absolute neutrophil count; BSI, bloodstream infection; IQR, interquartile range; PS, propensity score; SMD, standardized mean difference.
